# On Improving the Condition of the Insane

**Published:** 1851-10-01

**Authors:** Henry Monro

**Affiliations:** Fellow of the Royal College of Physicians, Author of "Remarks on Insanity, its Nature and Treatment," &c.


### ON IMPROVING THE CONDITION OF THE INSANE.

### PUBLIC ASYLUMS FOR THE MIDDLE CLASSES.

BY HENRY MONRO, M.B. OXON.

*Fellow of the Royal College of Physicians, Author of "Remarks on Insanity, its Nature and Treatment," &c.*

It is but too well known that the present means available for the treatment of the insane are insufficient, and that those which exist offer much room for improvement. But if one class of lunatics, more than another, seems to call peremptorily for relief at the present day, it consists of those who are sprung *from respectable but poor families.*

For the wealthy all conveniences are open,—whether private asylums, lodgings with medical men, or their own houses; and it is the fault of their friends more than their circumstances if all is not done for them which can be done.

For the poor, or labouring classes, the county lunatic asylums, the hospitals of Bethlem and St. Luke's, &c., afford great and suitable accommodation. The economy and diet of these houses is perhaps superior to anything which they have hitherto enjoyed; the degree of respect with which they are treated equals what they have always received from and given to their companions; and the social habits of the patients are (setting their maladies aside, in which they are common) not inferior to those which they have always known.

But what is there for persons of habits as refined as their richer neighbours, and education often superior? They cannot afford the former alternative, and are too often compelled to accept the latter, and this at a cost which none but those who witness their sufferings can at all appreciate.

What can the father of a family, the possessor of an income averaging

150*l.* or 200*l.* per annum, do, when one son out of five becomes insane? or what can the children do for that father? What can the clergyman, the medical man, the man of small business—I may say the great majority of the middle classes—do? Can those who have always known what it is to have a home of refinement, though not of affluence,—who have been accustomed to the quiet and affection of that home (often all the more tenderly regarded because poor),—be thrown among the illiterate and coarse-minded, and escape with impunity, not only injury to their present feelings, but, what is worse, a great obstacle to their chances of recovery? Would it not be dreadful to a person in health, living the life of a gentleman, if he were thrown suddenly into a workhouse, and treated like a pauper,—if he were not only separated from all the ties of affection (which in the case of the insane may be necessary), but surrounded suddenly by that which he has ever looked upon as a resource which, under no circumstances, he could accept? And if this is the case with a person in health, who sees and knows the boundaries of what is and what is not, who can exercise his intellect in contriving plans for himself, and knows that all human evils have their limits,—how dreadful would it be to minds which are oppressed with that anguish and restlessness which only those experience who have lost the power of their judgment, and know not whither they are drifting! But more of this by and by.

Before I continue the discussion of the necessities of the case, I would state briefly the sort of remedy which appears to me to be needed. The plan which I would suggest is similar in its general object to those which philanthropic men have suggested before—viz., *the establishment of Asylums for the Middle Classes.* Its only claim to originality is, that it differs in the details of commencing this system. But this difference is the more insisted on, from the belief that the former schemes failed, not from the want of sufficient object to attract interest, or of benevolence of intention to excite sympathy, but from attempting too much at first, and attempting this on a plan which hardly seemed to meet the exigencies of the case.

Plans have, I believe, been suggested to build a large hospital for the class which I allude to, and to charge some such sum as 100*l.* per annum as the minimum of reception into such a hospital.

The reasons why I object to the details of such a design, while I approve of the intentions of its organizers, are—*First:* That to attempt to raise a large sum of money for the establishment of an object which is only in embryo, has the character of incapability too much stamped upon it. How difficult it is to raise a large sum when the object and scheme are well known and appreciated,—for an ordinary hospital, for instance (a mode of charity more trusted by Englishmen than any others); how much more difficult, then, must it be when the advantages of the scheme are only imagined by a few, and known as facts to none. *Secondly:* To charge a sum approaching 100*l.* per annum as the minimum of reception into the house, in point of fact excludes the class of persons for whom I am interested,—for how can persons of small incomes afford so large an amount?

Let us begin at the other end. Let the work grow according as it proves its worthiness; let us trust to the devotion of the few who are willing to organize it, before we appeal to the sympathy of the public, who are at present uninterested. If those deeply interested in the matter would begin in a sure though humble manner,—would set a precedent and show an example,—much might be rightly expected and certainly achieved.

Let us suppose we could open a house for forty inmates (twenty of each sex), and place the expense of opening the house on the charity of a few individuals, and the current expenses of each inmate upon his own friends. Let us consider that each inmate would require the expenditure of 50*l.* per annum,—namely, 40*l.* for his keep and share of attendance, and 10*l.* or his share of house-rent, coals, and candles, &c.; that the 40*l.* per annum were defrayed by his relatives, and the 10*l.* per annum represented the gift, —the medical attendance being the voluntary work of such gentlemen as were interested in the working of the house.

I put this forward as a sketch of a scheme which might be commenced as an experiment. I by no means imagine it to be the best that could be devised, or free from objections: I know that many will think that I reckon the expenses at too low an average; but if they consult the averages of hospitals generally, I believe I shall be found not to be too sanguine.

Families in the receipt of from 150*l.* to 200*l.* per annum could and would give what is not much more than sufficient for the support of their sick brother or sister under any circumstances, though they are not able to afford him such luxuries (or rather I would say necessaries) as *privacy, comfort,* and *skill.*

Now if it were possible to open a house for forty inmates at the rate above mentioned, those who organize the charity need only feel themselves responsible for 400*l.* per annum,—a sum which five or six gentlemen interested and concerned in the matter would not, I imagine, have much difficulty in raising. For this sum the house could be opened, the scheme started, and, I trust, a precedent set of a most important and useful nature.

The size of this establishment would not, of course, be equal to the wants of London, even if we exclude all those above and below the class which interests us at the present moment. But it would occupy no inconsiderable position. For, if we reckon the length of residence of each inmate at 14 weeks (not an unfair average for acute cases, and acute cases are the only ones contemplated), by having forty inmates continually succeeding one another, we open our doors to about 150 cases in the course of the year. Care, of course, must be taken that no inmate should continue to reside when his case has become chronic; and therefore some period between six months and one year should be fixed upon as the extreme limit of residence under any circumstance.

To enter further into the details would be inappropriate on this occasion; each person can judge for himself of its capabilities, if conducted with untiring energy and kindliness of heart.

It will be said there are many private asylums willing to receive patients at 40*l.* per annum; but the melancholy and ready answer is, that out of this sum a livelihood is obtained or a fortune made by the proprietor. This fact speaks volumes. I will only add, that one main characteristic of what I would propose should be, that no private inducement should be held forth to any one to take a share in the scheme beyond the pleasure of being at work in a just and generous cause.

I will now enter more in detail upon some of the chief arguments which induce me to advocate the scheme.

And, 1st, I would urge some of the arguments for the general scheme; 2ndly, those for the peculiar mode of carrying it out, which I have mentioned above.

### Section I.

*On the general necessities for some such scheme as that suggested.*

My arguments for this description of charity divide themselves into two heads:—

I. The necessities of the class contemplated.

II. The general advantage likely to arise from adopting *public* measures for the treatment of the insane of the upper classes.

I. *On the matter of the necessities of the class, I would urge—*

1. That we frequently witness the grievous distress arising from the want of such means, and that the reason of this distress is manifest enough.
2. That the class for which I plead have, independently of the urgency of their distress, peculiar claims on the attention of those who feel for the sufferings of their fellow-creatures, inasmuch as that they have received hitherto peculiar neglect.

1. As an example of the wants of this class, take such cases as the following, which I select, not on account of any remarkable circumstances about them, but because they have occurred lately, and are fresh in my memory:—

A gentleman, an inmate of a private asylum, where he received and appreciated kindness, was, the other day, suddenly removed to St. Luke's, from the want of means to continue his residence elsewhere. I saw him continually while at the first-mentioned asylum; he seemed contented with his lot, conscious that he was wrong in mind, and sensible that the treatment he received was kindly meant, and equal to what he had any right to expect in his then existing condition. The head attendant, who took the gentleman to St. Luke's, told me, that it was a most distressing sight to witness his misery when he found himself suddenly herded with a class necessarily very inferior to his own; he told me that the patient wept like a child, and "that it was one of the bitterest sights he had ever witnessed."

A gentleman who had been in confinement for many years understood that he was to be removed from the private asylum in which he was, to a pauper establishment, on account of the lack of means of his friends. This person lay awake for nights in great distress of mind; he would prostrate himself at my feet, imploring me to interfere: his anguish appeared to be truly pitiable. This gentleman was not removed: indeed, the whole circumstance had arisen out of a mistake.

Another gentleman, whom I saw a few days ago, is now wandering about, and living a most wretched life of anxiety and fear, as well as danger to himself and others, because his friends cannot afford a private asylum, and cannot make up their minds to commit him to a pauper lunatic asylum. And I must say, that however much I saw the necessity for restraint, I could not but sympathize heartily in their hesitation as to adopting this the only mode at their disposal.

There has been, recently, a surgeon at Bethlem, who was for a long time under my care at a private asylum; he is now, or has been lately, classed among the worst patients there, being very offensive in his habits, &c. This gentleman is among society utterly incongruous with his former habits, and it is very painful to witness it, though in his case there was not that degree of sensibility which exists in many.

I trust it will not be thought that I imagine that the excellent institutions of Bethlem and St. Luke do not fulfil their work liberally and honourably, because I deplore the social condition of some of their inmates. These charities would go out of their proper sphere of action did they make distinctions of social ranks: all persons admitted within their walls come in the light of destitute persons who have no other available means of support or aid through their illness. That persons of higher rank in the social scale are frequently received is certainly the case: *but this is the very point I deplore.*

I will not add any more cases of distress arising from the want of asylums for the middle classes: it is wearisome to myself to write them, when I know how common they are: how much more wearisome must it be to read their common-place record! I only bring these few forward as instances of ordinary life.

But there are two arguments likely to be raised against the importance of this mode of charity, which I would rebut in this place: it will be said by many, that *the wants of this class are of too refined and unreal a nature to elicit a charitable work;* and, *secondly, that the insane are incapable of appreciating even these refined desiderata.* I will say a few words on these two points separately. And

*First, I assert that the ordinary history of life shows us that the absence of the refined courtesies and habits of life is not a trifle.*—Those persons who argue, that the forms of society are trifles,—that to feel bitterly the necessity of communing only with those with whom we not only have no sympathy, but who wound our feelings, however unconsciously, at every turn,—is a refinement of sensibility which charity cannot be expected to provide for,—would, I confidently believe, argue very differently if they themselves had experienced the like disruption of what they had esteemed most precious. Does not ordinary life teach us this lesson! the poor gentleman willingly starves before he can make up his mind to enter employment where all his associations must be grievously injured; the poor labourer allows himself to go through every suffering with cheerfulness before he will be degraded to pauperism, and what is not much better than prison discipline. Even when life is departing, and when our dying brother or sister has long ago taken leave of its hopes and fears, we still find the power and cogency of the instinct which drives a person to cling to the very last to the little refinements of life deemed to be so essential to self-respect.

These refined sentiments are, to the man of cultivated mind, more essential than food to the hungry or clothing to the naked; and, however much the utilitarian may say, that "if a man prefers starving to degradation, I cannot help him, he must starve,"—however much those in authority may hope, with all kindness of intention, to intimidate such an one into accepting relief which obliges the relinquishing the refined emotions of life, nature will not change,—he will go through dreadful sufferings before he will yield.

And it is good that this should be so, if refinement and cultivation have any real worth,—if the grosser element is not always to master the more ethereal, and if our delight in the objects of our senses is not always to overcome our delight in those more abstract operations of the mind by which man's position is so pre-eminently discernible from that of the lower animals of creation. Sad, indeed, would it be for us, should the day ever come when the voice that pleads for refined sensibility is not heard to speak with thrilling and convincing accents,—when associations are esteemed dreams, and only the grosser desires realities.

But, secondly, it may be argued that the insane do not feel the influence of these refined associations, and do, in consequence, not regret their absence. This is true of certain classes of the insane, but the exact reverse of this is the case with certain other classes,—the classes I am interested in, namely, those of incipient and active insanity. The imbecile may not feel them; those who have suffered shipwreck and lie stranded unconscious on the barren shore of this most fell disease,—may be and are dead to such considerations; but he whose vessel is hurrying to that shore, who sees his fate, struggles with it, and yet is spell-bound, feels all these things to a most morbid and exaggerated degree. In the stages of insanity before the imbecile stage, things are often exaggerated into importance which appear to have no importance to the healthy; an unguarded expression of an attendant is thought to mean something very terrible! a word of disrespect an insupportable insult! On the other hand, kindness of manner and gentleness of tone seem (according to the revelations of those who recover) to shed a brightness more brilliant, and more significant with meaning and intensity, than the sane can even imagine.

It is a great mistake to *think that confusion of thought confers obtuseness of sensibility: just the reverse is the case. It is a great error not to distinguish clearly between active madness and imbecility.* For be it ever remembered, as a great and important truth, that confusion of mind increases mystery, and mystery *ever* increases emotion: may I not almost add, that clearness of head dissipates keenness of feeling? For the truth of these observations let us again look into the page of ordinary life: all men suffer from delirium at times: for what is dreaming but delirium, and what is madness but such a state of our nervous organism going on in the waking state as ordinary persons only suffer from in that state of vital depression which we call sleep? Now do we not know by experience that the revelations, the images stamped on the mind in dreaming, are more keenly felt than *the same* would be in the waking state? Thus, a little bodily ailment in sleep leaves the impression of a terrible disease, conveying such a sense of woe as we hardly ever realize when awake: painful images produce in sleep horror which, if revealed to us when awake, might cause fear and anxiety, but no such sense as that of nightmare. Indeed, the emotion which we call horror, and which is so very terrible, hardly seems to belong to the economy of a cultivated mind when awake and in health: *is it not peculiar to the imperfect states of childhood, dreaming, and disease?*

Now, the reason why confusion and mystery should so increase emotion, and why clearness of head should dissipate it, is sufficiently manifest if we analyze our mental condition in this life: for we are in sight of good as of a vision ever fleeting, and yet have a constant struggle to preserve ourselves from evils, both physical and moral, which are ever forcing themselves upon us; and our chances of maintaining our ground in this struggle depend on our intimate acquaintance with the vicissitudes that surround us. We know this *instinctively*, and in consequence proportionably desire accurate knowledge. When a person is in health of mind and body, he is clearly conscious of the position that different objects bear to one another; he knows what to expect next; to what an extent things are likely to go; he knows their boundaries; and, much more than this, he knows that there are boundaries: whereas in disease all this is lost. Thus, in the sane state, we can clearly distinguish between objects of sense, objects of abstract thought, our own identity, and the relation that these several distinct matters have to one another. But how is it in delirium? We lose sight of all these divisions; we think an object of sense is a horror of mind, a dyspeptic twinge, a sense of mental woe. We do not perceive clearly our own existence and individuality: thus, as in disease, we may imagine that some little pain of our own intimates that some one else whom we love is going through great distress, and that we are only tortured by sympathy and the inability to help them. Abstract thoughts, again, are esteemed terrible bodily sufferings,—all power of distinction, and of course all power of discerning the relation between the various objects of consciousness, is lost.

How often may we observe that the person in delirium lies and watches one sitting by his side as if his attendant's countenance were the index of mysteries unfathomable; anything he does is thought to have a wretched air of indefinitiveness and inconsistency. Again, how the patient sighs after the countenance which he loves with a restless desire which is truly affecting, and out of consonance with the ordinary economy of life! We pass by the expressive gestures of the poor madman,—we content ourselves by saying it is all disease; but to him who desires to read the history of man's life and suffering, there is a page lying open to him which reveals more than the writer of fiction could conceive.

But to proceed. Because I dwell so much as I have done on the sensibility of the insane, and desire so much to mitigate their sufferings, let it not be imagined that I am not as practically aware as any one of the necessity of doing violence to many of the tender associations of the insane, and preventing them from having their full swing. For instance, I know that frequently the mind of the insane cannot be brought under that control which is necessary for a cure, without removal from friends and home; and I know this, not only from my own and others' experience, but from the confession of patients themselves when they recover. But because I see that sensibilities must be thwarted, I by no means think that habits which conduce to self-respect should not be studied, and disrespectful treatment from others vitiates it. The preservation of self-respect is conducive to strengthening the mind, though the encouragement of highly wrought emotions is injurious.

The advantages of asylums, however, are too obvious, and have been too well discussed, for me to comment upon them in this place. These external means seem to supply that support which vigour supplies to the mind in health,—they alone can restrain propensities which reason and judgment no longer can control. Notwithstanding that it is very natural to imagine that the presence of other insane people must do more harm than good to those already ailing, experience seems to show the converse. Frequently, a patient will (so far as he seems capable of moral influences, and is not entirely the victim of his physical disease, and the majority are in this state) feel the necessity of checking himself when he sees to what propensities similar to his own lead; he will feel hope rather than misery when he sees lower depths of misery than his own. This of course depends on constitutional temperament, whether it is elastic or the contrary, but still this is frequently the case.

2. Taking it for granted, then, that patients do feel deeply the want of such asylums as I now advocate,—a fact which others can substantiate as well as myself by many instances, and allowing that this is a result likely to be expected, considering how deeply the refined associations of life are generally felt, and how peculiarly certain classes of the insane feel these things,—I would urge as a second reason for attending to the want of this class, that they have hitherto had less done for them, less sympathy shown them, and heavier burthens thrown upon them, than either those above or below them. I quote the words of one who has seen much of this class:—"In the majority of cases the daily bread of the middle classes is as much dependent upon the mental and bodily vigour of the head of the family as that of the mechanic or labourer. Deprivation of reason for any lengthened period may be called the certain prelude to the decay and impoverishment of the family. Deprived of the exertions of its head, the family (even if able to struggle on) is unable to afford the cost of sending him to a private asylum, or only able to send him to such an one as tends rather to increase than cure the malady. He had better go to a well-regulated county asylum, so far as treatment is concerned; but to this the feelings of honest pride and self-respect on the part of his relatives generally demur."

How pitiable soever the scenes of the lowest grades of distress are, they are not more heart-rending than those of the class which is struggling to maintain a respectable footing, and sees by unavoidable illness what they have ever enjoyed fast fleeting away. This strange contrast damps all the energies of life far more than a biting poverty, which has become habitual. The workhouse-boy has hope, because he starts from the lowest ground, and can only ascend; you see his spirit elastic, his countenance cheerful. But the beggared child of respectable parents looks differently: dreams of the past are stamped indelibly upon his mind; habits have been formed which cannot be forgotten. The winter wind, which urges the little beggar to more vigorous gymnastics, creeps into the very soul of him who has known it only as it sounded in former days in his once comfortable home. I say not this becanse I wish to palliate the terrible sufferings of poverty, or because I agree with those who blandly say that paupers are used to it, and so it does not signify. I have seen too much of poverty to argue thus; but I say it to show that there are sufferings far worse than those of simple physical want, and that the sufferings of this class are as worthy of our regard as those of the classes beneath them.

But supposing the distress of the two classes to be equal; this class in a certain sense *deserves more at our hands than the poorer class.* For though pauperism is by no means always the result of sin, it very often is so; and on the other hand, to have maintained a family with difficulty in an educated and respectable condition, is itself very often a sign of good intentions and good living. Wealth and realized property may argue little as to moral position,—not so with hard-earned respectability; and yet we have assisted, and are continually assisting, the poor, while we have done little or nothing for the class just above them. Education is a talent which each person is bound to accept when offered to him; and yet, when it is attained, it doubtless very much increases sensibility to all the evils to which we are subject.

Such are some of the arguments by which I would plead for the class of poor but respectable persons. Did space permit, I could doubtless add many more.

II. This mode of charity will be very useful in another way than the direct good that it will do to the parties immediately concerned: I mean, that it will assist in rendering the treatment of the insane a public instead of a private matter.

There is a dilemma connected with the treatment of the insane, which renders progress difficult and dangerous: it is, that all immediately connected with the insane desire (and very properly so) to keep the disease private; while the helplessness of the patient, and the peculiar nature and consequences of his affection, often stand in need of public guardianship. The history of the treatment of insanity, as it stands contrasted in old and later times, reveals this truth unmistakably.

Thus two interests are raised. The one, the necessity of secrecy for the good of the family, as well as for the patient's own good (when he comes out into the world again); the other, the good of the patient under his immediate sufferings, should he happen to fall into untrustworthy hands.

Private motives (whether of the friends, or of the patient after recovery, or of the medical gentlemen and others who are paid for the reception of such patients,) are amply sufficient to guard and supply the first of these interests. Private asylums, in consequence, flourish, and will flourish, unless the evils to which they are prone should prove irremediable; which is not likely to be the case, for all connected with private establishments know (if their own consciences are not a sufficient guardian of their interests and actions) that the public keenly examines all their acts, and that in our country reform will come sooner or later, when change does not do more harm than good.

But the public are peculiarly the guardians of the other interest; and I am bound to substantiate what is already well known, namely, that the sort of asylum contemplated in these remarks will assist in doing away with some of those abuses which the private system has encouraged.

One evil, which has been inseparable from the private system, is, that it has engendered rivalry rather than community of interest among medical men connected with the insane.

Thus the profession has too often been degraded to a trade, rather than exalted to a science. This fact has been much felt of late,—it has given rise to many unwarrantable aspersions, and also well-founded suspicions. Now the establishment of asylums for the middle classes will be an additional source whence more liberal principles may flow. Should we succeed in London, it may well form the precedent for similar exertions in the provinces; and if, as I shall hereafter propose, the care of such institutions is not given up to individuals, the interest and advantage of fellowship in doing good will be extended. This is, however, a trite matter by this time, and has been well discussed.

But there is another point which appears to me to deserve much consideration, and which may have escaped equal observation: it is, that that neglect which the friends of patients used to evince towards their suffering relatives appears to decrease yearly, and to become obsolete in proportion as the treatment of the insane becomes a matter of public interest and care. If we compare the interest felt now-a-days by friends and relations with that which used to exist, it will be striking enough.

Now this improvement on the part of friends in recent cases, may be accounted for in various ways. In the first place, recent cases have more of hopefulness in them than old cases. For persons to continue to take a deep interest in relations, ten, twenty, or thirty years imbecile, is almost more than can be ordinarily expected. Secondly, persons in the present day begin to take a more enlightened view of the disease; they cease to look upon it as a demoniacal possession,—a fault of mind rather than body,—and view it rather in its pathological aspects, as an infirmity to excite pity rather than a fault to cause disgust. But beyond these reasons, I cannot but think that public opinion on the subject has its influence as it has in all other things; and that the increase of public feeling on the subject, and the decrease of the possibility of privacy, have each of them a strong influence in the result. It may seem strange that any other motive should be required but love and innate shame to make friends attentive.

In all other diseases, as well as in most of the evils to which we are subject, we can trust to sympathy and pity, at least in families where there is any regard for what is right: and to intrude other motives would be as injurious as unneeded. But in the terrible instance of insanity, sympathy and shame seem sometimes to have reached their boundary, and self-defence occupies their place. Let us compare insanity with some other ailment—say consumption. In the latter case we know, by happy experience, that dying hours are soothed, and dying brethren loved more than ever. In the former, we find that the hours of delirious pain and anguish do not receive even that attention from relatives which medical treatment permits. Why is this? I have already answered it by saying, that sympathy and shame have found their boundary. *Human love, in these cases, is not sufficient for the crisis, and public philanthropy is needed.* In consumption, sympathy, so far from being blighted, grows day by day. The pain of waiting and watching is more than compensated by thoughts which have a most personal application. We, too, must die, and our body slowly decay: we want to learn how to die, even in the midst of life, and here our lesson is given; we want to have aid ourselves when objects grow dim before our fast-fleeting sense, and by this means we acquire a right to expect it. We keenly desire to watch one who has always been dear to us, growing more precious as the hour of departure approaches. The feelings are refined and sanctified, but still pleasure is attained rather than unalloyed pain. How changed is the case in insanity! We dread to look at one we have loved so horribly changed; it causes a revulsion to all our aspirations,—a shock we cannot stand: we do not expect to lose our own mind before our body dies, and therefore we do not think of acquiring a right for kindly treatment for ourselves; we do not want to learn how to suffer madness,—the need does not ordinarily exist. Thus our sympathy is as much excited in the one case as it is terribly checked in the other; and, placing sympathy aside, we find that even the baser motive, shame, in some cases, is not sufficient for the crisis; then the sense of public indignation is useful. In consumption, we know that all which is done and said is noted down by the sufferer and those who watch with us; but in insanity, the pleadings for more of care and attention are too often looked upon as the mere utterance of delusions; and the selfish escape under a thick cloak, which is not removed, perhaps, while life lasts. Shame, moreover, takes another form of action, and the friends are (with some degree of plausible excuse) more ashamed of owning than of neglecting their relations,—more ashamed of the stain of the disease being in the family, than the stigma of not visiting them as much as they ought.

I have a happy experience of more noble conduct, and therefore far would I be from laying a charge on the relations of the insane generally. I would make it the exception now rather than the rule; and I am free to confess, that instances of a directly opposite nature are common—namely, where old affection survives and flourishes through scenes of a most revolting nature, and that even in cases where moral depravity has more concern in the matter than physical defect.

### Section II.

*On the advantages of the particular scheme suggested.*

Among other arguments which might be raised against the specific form here proposed for working the Asylum for the Middle Classes, it will no doubt occur to many, that in proposing a new institution it is unadvised to make it partly self-supporting and partly a work of charity, as we appeal to no one passion sufficiently;—that it is not sufficiently a charity to excite the lover of good works, nor, on the other hand, sufficiently business-like to attract those who are chiefly interested in a self-supporting or paying institution.

My answer is,—1st, that if it were wholly a charity, it would not suit the class I am advocating; 2nd, that if it was wholly a marketable business, the effort would not be likely to succeed, from the fact of its novelty and untried difficulties. I would therefore appeal to the genius of charity to open the house, and to the genius of business to come to her share of the work in due course of time.

1st. If it were wholly a charity, it would not suit this class. *They would be either too proud to accept it, or obtain more aid than they deserve.* The high-minded of this class,—the poor clergyman, medical man, man of business, &c.,—would not endure the idea of their friends living on alms, and eating the bread of others. But this scheme allows their actual maintenance to continue at their own expense as usual, and their medical treatment to be the gift of those who receive back again by the opportunity offered for the study of the affection: so that all which remains as a gift is the expense of lodging, the obligation for which might be mitigated by subscription to the funds of the institution, or advocating its cause with their friends,—and these conditions are surely such as any open-hearted and generous man might easily receive without indignity. Again, if it were wholly a charity, this class would receive more than they actually are in need of. Heads of families possessing 150*l.* or 200*l.* per annum might and should afford 40*l.* per annum for a sick member: they must support him any how at a greater expense than the healthy members of their family, and at a cost something like this, though 100*l.* or 200*l.* per annum (sums which are properly enough expected in private asylums) are wholly inadmissible here. No doubt there are many good beggars, with incomes such as I have described, who would receive willingly *all* in charity if they could, and cast their suffering relative into the hands of others; but it is not our object to encourage this mode of proceeding.

2nd. To attempt a self-supporting institution at once would, I fear, fail of success: it would not, at present, ensure public support,—the public would feel that their armour had not been proved, and that they had to cope with great difficulties. We must have a more than ordinary spirit at work to start what ordinary strength may well continue.

I by no means desire that the scheme should not gradually become an entirely self-supporting one; indeed, this would be its most healthy end, and I trust, in consequence, that such would be the case; but it is one thing to prepare and bring the stone to the verge of the hill, and another to keep it rolling when once set in motion. Men of business well know that schemes continually succeed after a time, though the organizers of them are unequal to the task; and that a spur is wanted at the beginning which is not needed afterwards.

In fixing the sum of the whole expenses of each individual at 50*l.* per annum, I am aware that I shall be charged by many with making it too small, considering that washing and attendance are included in this charge: but I do not speak without consideration and experience. I am intimately connected with a charity, where respectable poor persons are received, who have been thrown out of employment owing to no fault of their own, and who are received within the walls of this refuge in order that full employment may be found for them by the gentlemen who visit the institution, and that their strength may be restored by good and ample diet. The inmates of this charity have meat every day as much as they want (except on one day when they have fish and soup); their diet in other respects is liberally conducted, and on the principle of restoring their strength, which has been previously exhausted by privations; and the average expense of this institution (which contains between 40 and 50 inmates,) is only 5*s.* a week per head in food; about 15*l.* a year per head. This has been the case for some years now, and *is a good example of the union of economy with liberal allowance.* I grant that in the first year or two the diet was more expensive, though not better; but this was simply the fault of want of experience and good superintendence. I could add many more arguments in favour of the scheme which I have proposed, but refrain, from the belief that I may easily become tedious, and the desire not to appear, by hedging it round with defences, to view this scheme as by any means perfect. All that I wish is, that something of the sort should be attempted; and I wish that my suggestions may be added to those which have been urged on the same subject before, and with greater ability.

Before I conclude, however, I would make one or two general observations.

I esteem it to be very necessary that four or five medical gentlemen, of experience and reputation, should be connected with the control of such a charity; not only that by this means gratuitous medical aid may be given with ease, by the division of labour, but more especially, that this would be the only safe mode of removing the charge of deriving personal advantage from it. If one or even two conducted it, it might be said that such a charity had become but the feeder of their individual private practice (a common and not unnatural charge urged against medical charities). Moreover, it would lose the great advantage of being a means of uniting those connected with the care of the insane. Any honourable mind engaged in this branch of the profession would feel the weight of these charges peculiarly: and they would very rightly deter a man from entering the arena. We may stand with impunity many aspersions, but *this sort* of charge is one which all should desire to escape from, both for his own well-being, as well as that of the work in which he is engaged.

Another point is, that very rigid rules must be drawn up as to the choice of inmates. No doubt many would desire to avail themselves of the advantages of such an institution, who can well afford more expensive modes. These should be excluded. Those also whose position of life is at all suited to county lunatic asylums would of course be out of place here.

I am free to confess that the interest which has lately been excited in my own mind on this most important subject is so great, that I can well believe I view it as a hobby, and as a panacea, too much; still I must own that the more I reflect on the subject the more it grows upon me, and the more am I inclined to wonder that this idea, which has been contemplated so long by great and good men, has not been embodied into a reality. Should it ever exist, and should good results spring from it, it will only be another instance of how continually we are on the verge of finding a treasure and pass it by unconsciously. I say this the more urgently from the great dislike which I as an individual have to an undue dread of novelty. Our countrymen seem instinctively to be averse to what is new, and though another quality of their nature makes ample amends for this, causing them to go far ahead of their more volatile neighbours in the long run, *namely, resolutely adhering to a change which they have once proved to be good*, I must own they often deal unjustly with a matter, simply because they have not tried it, and will not do so.

But I have said enough: I will only add my conviction that should such an institution be contemplated, those concerned in it must unite with a more than ordinary zeal and good-will in carrying it into execution.

